# Disease awareness in myotonic dystrophy type 1: an observational cross-sectional study

**DOI:** 10.1186/s13023-016-0417-z

**Published:** 2016-04-04

**Authors:** Sigrid Baldanzi, Francesca Bevilacqua, Rita Lorio, Leda Volpi, Costanza Simoncini, Antonio Petrucci, Mirco Cosottini, Gabriele Massimetti, Gloria Tognoni, Giulia Ricci, Corrado Angelini, Gabriele Siciliano

**Affiliations:** Department of Clinical and Experimental Medicine, Neurological Unit, University of Pisa, Via Roma 67, 56126 Pisa, Italy; IRCCS San Camillo Venezia, Via Alberoni 70, Venezia, 30126 Italy; Neurology and Neurophysiopathology Unit, San Camillo Forlanini Hospital, Piazza Carlo Forlanini 1, 00151 Rome, Italy; Department of Translational Research and of New Surgical and Medical Technologies, University of Pisa, Via Paradisa 2, 56126 Pisa, Italy; Department of Clinical and Experimental Medicine, Psychiatry Unit, University of Pisa, Via Roma 67, 56126 Pisa, Italy

**Keywords:** Myotonic dystrophy type 1, Steinert’s disease, Neuropsychological impairment, Disease unawareness, Quality of life

## Abstract

**Background:**

Myotonic dystrophy type 1 (Steinert’s disease or DM1), the most common form of autosomal dominant muscular dystrophy in adults, is a multisystem disorder, affecting skeletal muscle as well as eyes, heart, gastrointestinal tract, endocrine system, and central nervous system, finally responsible of increasing disabilities and secondary social consequences. To date, DM1-related brain involvement represents a challenging field of research. It is well known that DM1 patients frequently present neuropsychological disturbances and psychiatric comorbidities among which reduced awareness of disease burden and its progression, also defined as anosognosia, is common in clinical practice, this leading to secondary misattribution of symptoms, delay in timely diagnostic procedures and low compliance to treatment.

**Methods:**

Here we present an observational cross sectional study in which disease-related cognitive dysfunctions and quality of life were assessed by a protocol finally designed to estimate the prevalence of disease awareness in a sample of 65 adult-onset DM1 patients.

**Results:**

Our analysis showed that in DM1 patients several cognitive functions, including executive and mnesic domains with visuo-spatial involvement, were affected. The assessment of anosognosia revealed that a high percentage (51.6 %) of DM1 subjects was disease unaware. The reduced illness awareness occurs across different physical and life domains, and it appears more prominent in *Activities* and *Independence* domains investigated by the Individualized Neuromuscular Quality Of Life (INQoL) questionnaire. Moreover, the unawareness resulted significantly related (at *p* <0.05 and *p* < 0.01) to the performance failure in cognitive tests, specifically in the domains of visuo-spatial memory, cognitive flexibility and conceptualization.

**Conclusions:**

The obtained data confirm, by a systematic analysis, what’s the common clinical perceiving of disease unawareness in Steinert’s disease, this related to the already known cognitive-behavioural impairment of frontal type in affected patients. We believe that a deep knowledge of this aspect will be useful for medical practice in the management of patients with DM1, also for guidance in occupational and social interventions, definition of outcome measures and in preparation of trial readiness.

## Background

Myotonic dystrophy type 1 (DM1, Steinert’s disease) is the most common form of muscular dystrophy in adults, with a prevalence of about 1 in 8,000 people worldwide. It is an autosomal dominant disorder due to an unstable cytosine-thymine-guanine (CTG) triplet repeat expansions within the noncoding 3’ untranslated region of the myotonic dystrophy protein kinase (*DMPK*) gene on chromosome 19q35 [1–3]. The mutation causes mis-splicing of mRNA species which can affect many cellular processes in different organs and tissues. There is not always a clear correlation between phenotype and genetic repeat size [4]. In general, longer CTG repeat expansions are associated with an earlier age of onset and more disabling disease. Based on age at onset and clinical expression, DM1 can be classified into three clinical forms, congenital, childhood and classic or late-onset [5, 6]. Children with congenital DM1 present hypotonia and severe generalized weakness at birth, often with respiratory insufficiency and early death; intellectual disability is also frequent. Childhood and classic DM1 forms, ranging from mild to more severe phenotype, can be considered as a multisystem disorder, that affects skeletal muscle as well as eyes, heart, gastrointestinal tract, endocrine system, and central nervous system (CNS), finally responsible of an increasing disability and secondary social consequences.

To date, DM1-related CNS involvement represents a challenging field of research. Several CNS imaging studies have documented brain abnormalities in DM1 patients. Routine brain magnetic resonance imaging often shows non specific pathological findings such as white matter hyperintense lesions, ventricular enlargement and brain atrophy in temporal and frontal lobes, brainstem nuclei, thalamus and basal ganglia [7–11]. Single photon emission computed tomography perfusion imaging or 18 F-deoxy-glucose positron emission tomography demonstrated the presence of hypoperfusion/hypometabolism in frontal and temporal lobes [9, 12, 13]. Moreover, several neuropathological evidences confirmed a diffuse cell loss in specific areas of the brain, neuronal eosinophilic inclusion bodies in the thalamic nuclei, substantia nigra and caudate nucleus, neurofibrillary tangles of the type seen in Alzheimer’s disease and other neurodegenerative disorders in the hippocampus, entorhinal cortex and temporal areas [14].

Notably, in the last 20 years, several studies have well documented that DM1 is frequently associated with different neuropsychological deficits and psychiatric comorbidities.

### Cognitive functioning

Intelligence assessment documented an intelligence quotient (IQ) below normal for age in the DM1 population as compared to healthy subjects, with no clear evidence of a progressive intellectual decline [14, 15]. Several studies have demonstrated that DM1 patients show selective impairments in cognitive functioning, particularly in attentive, visuo-spatial, and executive domains [16, 17]. In 2004 Modoni and coworkers [18] strengthened this issue by studying a large cohort of patients stratified by CTG size: the authors demonstrated that visuo-spatial deficits are recurrent features in the neuropsychological profile of adult patients with DM1, regardless the size of CTG expansions. More recently, the concept of a “DM1-related-dysexecutive-syndrome” has been proposed to define the heterogeneous neuropsychological involvement in DM1 [14]. Nevertheless, it is still unclear whether in these patients there is a progression of cognitive decline over time [19], and whether the brain abnormalities evolve in dementia syndrome. Language function is generally preserved in patients with adult DM1 [12, 16], although speech abnormalities (dysartria) due to facial weakness or tongue/jaw muscle myotonia can result in difficulties in oral communication and social inclusion, with concern for patients and their families.

### Neuropsychiatric involvement

Beside cognitive impairments, in DM1 patients’ neuropsychiatric comorbidities are frequently reported with variable pathologic behavioral patterns. A high prevalence of dysfunctional personality has been described [16, 20], mainly in dependent, avoidant, obsessive-compulsive and schizotypal dimensions. In 2006 Winblad and coworkers [21] demonstrated that facial emotion recognition is also affected in DM1 patients, with some common features resembling schizophrenia, suggesting a possible similar pathophysiology. The occurrence of mood disorders is also reported [17, 22]. Affective and depression-like symptoms are frequently observed in DM1 subjects, whereas only few patients fully match the criteria for a depressive disorder [16, 23]. Lack of interest (apathetic behaviour), a decreased emotional participation and an increased irritability are common features, also defined as “an emotional imbalance” [24, 25].

### Disease awareness

In clinical practice, it is commonly noted that subjects affected by DM1 often show a reduced awareness of disease burden and its progression, also defined as anosognosia or lack of insight, this leading to secondary misattribution of symptoms, delay in timely diagnostic procedures and low compliance to treatment [14, 26]. The unawareness of disease can be observed in individuals with brain lesions or neurodegenerative disorders, such as Alzheimer's disease, in which it can be a direct consequence to the underlying pathological process itself [27]. Psychopathological mechanisms have been also hypothesized to be involved in the genesis of anosognosia. In our knowledge, to date, a systematic characterization of the occurrence of anosognosia and its correlation with the neuropsychological dysfunctions in individuals with DM1 is not available.

Here we present an observational cross sectional study in which disease-related cognitive dysfunctions and quality of life were assessed by a protocol finally designed to estimate the prevalence of disease awareness in a sample of adult-onset DM1 patients.

## Methods

### Study design

From December 2012 to December 2014, we consecutively screened 81 patients with clinical and genetic diagnosis of adult form of DM1, according the International Consortium for myotonic dystrophies guidelines [28], referred to Neurological Clinic of University of Pisa and S. Camillo IRCCS Institute of Venice. Patients with mental retardation (IQ < 70), severe visual impairment, psychiatric illness and a history of substances abuse were excluded. The final sample included 65 subjects (63 % males, 37 % females; mean age: 46.1 ± 12.3 years; age range: 18–70 years) (Table 1). None of the patients presented motor or coordination disability sufficient to account for possible delay in any of the neuropsychological tests administered.

**Table 1:** Clinical and demographic characteristics of recruited DM1 patients

Patients Enrolled	81
Drop-out	16
Final Sample	65
Age	46.1 ± 12.3 yrs
	Age range: 18–70 yrs
Sex	F = 37 % (24)
	M = 63 % (41)
Age at onset	31.5 ± 14.7 yrs
[CTG]n E1	37.7 % (25)
[CTG]n E2	58.5 % (38)
[CTG]n E3	3.8 % (2)
Transmission	78 % paternal
	22 % maternal
Educational level	Mean 11.6 ± 3.5 yrs
Time from disease onset	Mean 10.2 ± 7.4 yrs
Severity of muscular involvement (MIRS)	Mean = 2.85 ± 1
MIRS = 1	7.3 %
MIRS = 2	36.4 %
MIRS = 3	27.3 %
MIRS = 4	21.8 %
MIRS = 5	7.3 %

A group of 26 sex-and-age-matched healthy control subjects was also recruited. The study was authorized by the Pisa University Medical Ethics Committee (CEAVNO, North-West Tuscany Ethical Committee) and it was conducted according to the principles expressed in the Declaration of Helsinki; participants were asked to fill in a written informed consent form, in which they state that they have been adequately informed about the study procedures.

The experimental protocol was displayed into three examination sessions: a) comprehensive clinical evaluation, b) neuropsychological examination and c) assessment of quality of life and disease unawareness.

- The neurological examination included clinical history collection and evaluation of muscle involvement, scored by Muscular Impairment Rating Scale (MIRS) and Medical Research Council scale (MRC) [29]. All patients were sub-grouped on the basis of CTG expansion size (Table 1). A complete assessment of extra-muscular clinical features was also performed, including evaluation of cardiac, respiratory, gastro-intestinal, ocular and endocrinological involvement.
- Neuropsychological evaluation was performed in two phases; intellectual functioning was evaluated using Brief Intelligence Test (TIB), an Italian test for premorbid IQ estimation, in order to recruit only patients with IQ within the normal ranges. The first phase aimed to achieve behavioural characterization of DM1 patients. A psychological consultation was performed including psychometric assessment of depressive and anxiety symptoms, by Beck Depression Inventory-II (BDI-II) [30], State-Trait Anxiety Inventory-2 (STAI-Y2), [31], and of apathetic behaviour by Apathy Evaluation Scale (AES) (Table 2), [32]. In the second session patients underwent complete neuropsychological cognitive evaluation battery composed by: Immediate and Delayed Recall (IR, DR) of Rey Auditory Verbal Learning Test (RAVLT), Immediate and Delayed Recall (IR, DR) of Rey Osterrieth Complex Figure (ROCF), digit span and Corsi’s block test (CBT) to assess immediate memory, Trail Making Tests (TMT A and B) to assess selective attention and cognitive flexibility, Stroop Test to assess automatic response inhibition, phonemic verbal fluency test (FAS), Frontal Assessment Battery (FAB) and Modified Wisconsin Card Sorting Test (WCST), to assess frontal and executive functions, Rey-Osterrieth Complex Figure to assess spatial organization and visuo-constructional skills (ROCF), and Raven’s progressive matrices (PM47) to assess culture-free abstract reasoning [33, 34]. Patients’ raw scores were corrected according to Italian normative values (Table 3).

**Table 2:** Psychological characterization of DM1 patients by clinical scale administration (Beck Depression Inventory, BDI-II; State Trate Anxiety Inventory, STAI-Y2; Apathy Evaluation Scale, AES) BDI-II: total score ranges 0–63, normal ratings ranking below 9 for men and o13 for women STAI-Y2: total score ranges 20–80, threshold value = 40 AES: total score ranges 0–54, cut-off threshold = 18

	Mean (SD)
BDI-II (depressive symptoms)	10.6 (8.4)
STAI-Y2 (anxiety symptoms)	39.1 (10.0)
AES (apathetic behaviour)	18,2 (9.5)

**Table 3:** Neuropsychological functioning. Test scores are expressed as mean, (SD). Percentages of impairment *(% impaired*) are related to available cut-off scores of normality (≥95 % of the tolerance limit of the normal population distribution); statistically significant differences between patients and healthy controls are shown in bold

	DM1 patients (*n* = 65)		Controls (*n* = 26)	
	mean corrected scores	% impaired	mean corrected scores	*p*-value
RAVLT IR	42 (7.2)	5.8	46.8 (8.4)	**.013**
RAVLT DR	9.1 (1.8)	1.9	9.8 (2.5)	.370
Rey Osterrieth Figure (ROCF) Copy	27.5 (6.5)	46.9	32 (6.5)	**.007**
Rey Osterrieth Figure (ROCF) IR	13.8 (6.0)	32.7	19.8 (6.6)	**< .001**
Rey Osterrieth Figure (ROCF) DR	13.2 (6.7)	36.7	19.7 (6.4)	**< .001**
Digit Span	5.1 (1.1)	24	6.2 (1.2)	**.002**
Corsi’s Block Test (CBT)	4 (0.8)	43.1	5 (0.8)	**< .001**
Trail Making Test A (TMT A)	55.5 (34.9)	21.3	41 (14.3)	**.003**
Trail making Test B (TMT B)	130.5 (60)	17.4	108.3 (47.2)	.072
FAS (phonemic fluency)	28.4 (9.8)	25.5	44.7 (9.0)	**< .001**
FAB	15 (1.4)	25	16.3 (0.6)	**< .001**
STROOP Time Interference	32.8 (24.8)	32.7	18.9 (6.4)	**< .001**
STROOP Error Interference	3.2 (4.4)	36.7	1 (0.9)	**.001**
PM47	30.8 (36.4)	18.9	30.6 (2.3)	**< .001**
WCST Categories (Cat)	3.8 (1.9)	43.4	5.6 (0.8)	**< .001**
WCST Perseverative Errors (pe)	5.5 (4.4)	47.2	1.9 (1.2)	**< .001**
  Neuropsychological assessment was performed by an experienced examiner in a quiet and comfortable medical office. Each examination session lasted about 90 min.
- In order to investigate the disease subjective experience, we decided to use the Individualized Neuromuscular Quality of Life Questionnaire (INQoL) [35], a structured interview consisting of 10 items that capture patient’s physical and psychosocial limitations related to the muscle disease. Motor impairment is evaluated by items (1–4) referring to the impact of common muscle disease symptoms like weakness, pain and fatigue. Psychosocial limitations in daily activities are investigated by questions (5–9) referring to the impact of muscular symptoms on Life Domains, such as activities, independence, relationships, emotions and body image. Participants respond to the various issues using a seven-point Likert scale, thus allowing to obtain a patient-weighted score for each section [36]. Section scores and a total score are then calculated in term of percentage: a higher percentage is indicative of a greater negative impact on quality of life.

### Assessment of disease unawareness

Disease unawareness can be defined as an altered ability to recognize the presence or appreciate the severity of deficits in sensory, perceptual, motor, affective, or cognitive functioning and it has been operationally defined in a variety of ways. However, tools currently available for evaluation of disease-awareness are still under debate, since this dysfunction is closely related to the kind of illness to be investigated. In clinical practice, structured interviews, or questionnaires, addressed to patients and measures of discrepancy between main caregiver and self-reported clinical symptoms are commonly used [37].

To be eligible for the interview, caregivers have not to suffer from DM1 or from other illnesses affecting cognitive function.

In this study, patients responses to question asked about their functional capacities were studied and compared, and the following experimental protocol was specifically designed to evaluate the occurrence of anosognosia in each DM1 patient:

1. comparison between the severity of motor impairment scored by MIRS (administered by a trained clinician), and symptoms complained by patients and assessed by INQoL Weakness Domain (administered by a psychologist): we evaluated the statistical agreement between these two different assays.
2. administration of INQoL questions 5–9, regarding the Life Domains, such as *Activities, Independence, Social Relationships, Emotions*, both to patient and, separately, to the main caregiver, as already performed to test disease awareness in neurological disorders [37] the discrepancy score (D) was calculated as the difference between patients’ and caregiver’s scores (eg. *D (item 1) =* **caregiver score – patient score***)*. We considered the difference of 2 points or more in at least 2 items as indicative of the presence of a reduced disease awareness.

### Statistical analysis

Statistical analyses were carried out using the Statistical Package for Social Science (SPSS), version 21.0 and were performed by a medical statistician.

Demographic and clinical variables were compared between groups using the Pearson chi-square for categorical variables, Student t test and the Mann–Whitney U test. Cognitive scores were converted as means (and ranges) or frequencies (and percentages) of subjects with pathological scores.

The *p* value < 0.05 was considered statistically significant. All statistical tests were two tailed.

In order to investigate the relationship between clinical data and psychological variables, we computed the bivariate correlation coefficients, Pearson’s coefficient for parametric variables and Spearman’s coefficient for non parametric variables. An exploratory forward stepwise logistic regression analysis was carried out to determine which cognitive domains are best related to DM1 brain dysfunction; *group* (*patients vs controls*) was the dependent variable and TMT-A, TMT-B, WCST, STROOP, ROCF-copy, ROCF-IR, ROCF-DR and CBT as predictor variables, in order to detect the most affecting cognitive tests in DM1 patients. We performed Bland-Altman plot with 95 % limits of agreements for the combined graphical/statistical interpretation of patients’ self evaluation of muscular weakness (INQoL Weakness Domain score) to clinician’s objective muscular evaluation (MIRS).

## Results

### Neurological assessment

All patients were molecularly defined and grouped on the basis of CTG expansion size as follows: E1 (<150 CTG; 33.7 %), E2 (150–1000 CTG; 58.5 %), E3 (>1000 CTG; 3.8 %) (Table 1). MIRS scores varied between 1 to 5 (mild to severe impairment), with a prevalence of mild/moderate involvement (7.3 % of patients with MIRS 1; 36.4 % with MIRS 2; 27.3 % with MIRS 3; 21.8 % with MIRS 4; 7.3 % scored MIRS 5; MIRS mean value 2.85 ± 1) (Table 1). Mean time from disease onset was 10.2 ± 7.4 years. Patients’ mean educational level was level was 11.6 ± 3.5 years.

### Neuropsychological assessment

**Fig. 1:**
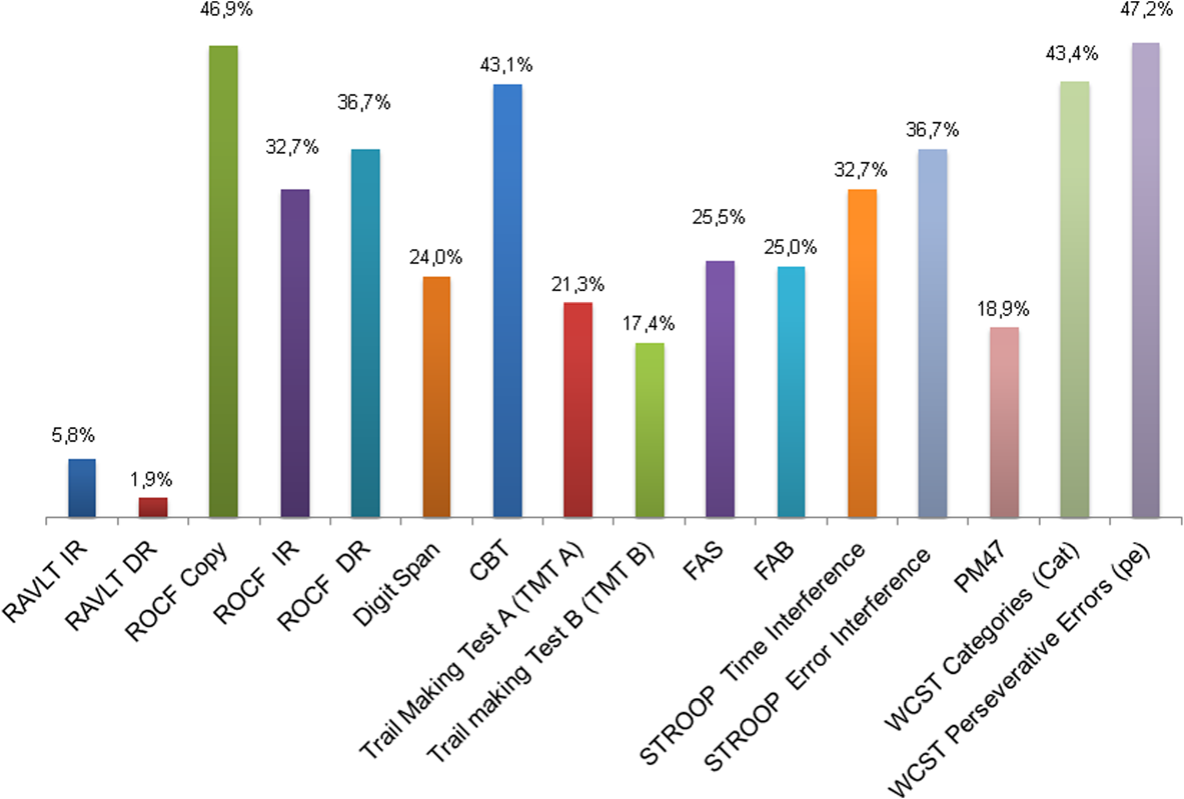
Percentages of DM1 patients who show critically relevant neuropsychological dysfunctions across different cognitive domains (*N* = 65). Percentages of impairment (%) are related to available cut-off scores of normality (≥95 % of the tolerance limit of the normal population distribution)

Overall, DM1 patients did not show severe depressive or anxiety symptoms as assessed respectively by BDI-II and STAI-Y2, while in these subjects there is a tendency to observe mixed mood conditions together with apathetic behaviour (Table 2)**.** Patients obtained lower scores than matched controls in most neuropsychological tests (Table 3). Notably, in patients we found that several cognitive performances resulted impaired (Fig. 1). Cognitive profile appeared heterogeneous, with no significant differences in cognitive performance between female and male patients. Main impairments were found in executive (Stroop, WCST) and mnesic domains with visuo-spatial involvement (ROCF-*copy* and *DR*, CBT). Interestingly, verbal memory abilities (RAVLT, Digit span) were rather preserved, suggesting a dissociation between verbal and spatial abilities.

Executive functioning was assessed using the specific cognitive tests previously described in Methods section. TMT A was impaired in 21.3 % of patients, TMT B in 17.4 %, FAB in 25 % and FAS in 25.5 %.

Impairments in Stroop test (Error Interference 32.7 %, Time Interference 36.7 %) and WCST (Categories 43.4 %, Perseverative Errors 47.2 %) were more frequently detected, pointing out the patients’ significant difficulties in engaging attentional resources to inhibit automatic behavioural responses, in using environmental feedback to shift cognitive sets, in directing behaviour toward the achievement of a goal, and in modulating perseverative responding. The high incidence of perseverations assessed in our sample suggest an inefficient initial conceptualization with consequent learning dysfunction across stages.

Immediate memory was tested with CBT for spatial performance and with Digit Span for verbal performance; 43.1 % of patients showed CBT impairment, while 24 % had impairment in Digit Span. As for delayed recall memory, only 1.9 % showed RVLT-DR impaired performances, while 36.7 % had impaired ROCF-DR.

Motor planning and visuo-spatial abilities, tested using ROCF, widely ranked below the normal range, with 46.9 % of patients with impaired performance. However, perceptual skills, assessed both by clinical and neuropsychological examination, were normal (Table 3, Fig. 1).

There was a decrease in cognitive performance related to age in the majority of the cognitive domains examined [RAVLT IR (*r* = −.348, *p* = .011) and DR (*r* = −.426, *p* = .002), CBT (*r* = −.423, *p* = .002), TMT A (*r* = .475, *p* = .001), TMT B (*r* = .559, *p* < .001),ROCF (*r* = −.445, *p* = .001), FAB (*r* = −.507, *p* < .001), Stroop time interference (*r* = .348, *p* = 0.14), PM47 (*r* = −.403, *p* = .003), WCST perseverative errors (*r* = .434, *p* = .001)], although data of follow up are not available in order to establish the degree of DM1-correlated cognitive impairment progression by aging.

It is important to note that the results of some cognitive tests could be impaired by the concomitant motor disability due to the muscle disease influencing test procedure’s and performance skills. For instance, within the men group the motor planning performance (FAB test, item number 3 consisting in Lurjia motor series) was affected by the presence of hand muscular strength impairment and myotonia, scored by MIRS (Spearman *r* = −.409, *p* = .025). Instead, among women, MIRS negatively correlated with visuo-constructive and spatial memory function, tested by ROCF-copy and IR (Spearman r_1_ = −.522, *p* = .046, r_2_ = −.583, *p* = .023).

The regression analysis was performed considering the most impaired test (TMT-A, TMT-B, WCST, STROOP, ROCF, CBT) as predictive variables and two test entered the model, ROCF-copy and CBT (Table 4). The model correctly predicted patients vs controls in 76.7 % of cases.

**Table 4:** Stepwise logistic regression analysis in 81 subjects (65 DM1 patients, 26 healthy subjects): CBT and ROCF-copy performances as predicting variables

STEP	Cognitive test	b (SE)	O.R.	C.I. 95 %	P
**1**	Constant	6.56 (1.78)	-	-	-
	CBT	−1.31 (0.38)	0.27	0.13–0.57	.001
**2**	Constant	8.19 (2.07)	-	-	-
	CBT	−1.21 (0.41)	0.30	0.13–0.67	.003
	ROCF-copy	−1.13 (0.05)	0.88	0.80–0.97	.007

STEP 2 R^2^ : Cox-Snell = .29, Nagelkerke = .40, Hosmer-Lemenshow: chi-square = 7.36, *p* = .498

percentage of overall correct prediction = 76.7 %

Moreover group comparison analysis showed that anosognosic patients had a higher impairment (*p* < 0.05) in visuo-spatial memory functioning (CBT), cognitive flexibility and conceptualization (WCST Cat, Ep) (Table 5).

**Table 5:** Group comparison between anosognosic (*n* = 33) and non anosognosic (*n* = 31) DM1 patients, in cognitive domains of functioning; statistically significant *p*-values are shown in bold

	Anosognosic (*n* = 33)	Non anosognosic (*n* = 31)	
	mean corrected scores (SD)	mean corrected scores (SD)	*p*-value
RAVLT IR	41.1 (7.8)	42.1 (6.9)	.612
RAVLT DR	9.0 (1.7)	9.1 (1.8)	.851
Rey Osterrieth Figure (ROCF) Copy	27.6 (6.1)	28.5 (5.7)	.597
Rey Osterrieth Figure (ROCF) IR	12.8 (6.3)	15.5 (5.5)	.124
Rey Osterrieth Figure (ROCF) DR	12.3 (6.6)	14.5 (6.8)	.265
Digit Span	5.1 (1.2)	5.2 (1.1)	.813
Corsi’s Block Test (CBT)	3.9 (0.8)	4.4 (0.6)	.060^a^
Trail Making Test A (TMT A)	54.1 (19.9)	57.8 (46.1)	.732
Trail making Test B (TMT B)	140.2 (78.9)	122.3 (31.4)	.351
FAS (phonemic fluency)	26.3 (8.7)	31.3 (10.5)	.083
FAB	15.1 (1.2)	15.3 (1.4)	.576
STROOP Time Interference	33.5 (28.4)	32.4 (22.2)	.881
STROOP Error Interference	3.4 (5.3)	2.5 (2.6)	.487
PM47	25.5 (0.3)	27.5 (4.2)	.202
WCST Categories (Cat)	3.0 (1.9)	4.7 (1.5)	**.001**
WCST Perseverative Errors (pe)	6.7 (4.6)	4.0 (3.4)	**.028**

^a^ considerable trend toward significance

Abstract reasoning, measured by using the Coloured Raven Progressive Matrices (PM47) was altered only in a small percentage of patients (18.9 %). Severe attention deficits were not detected.

### Evaluation of quality of life

**Fig. 2:**
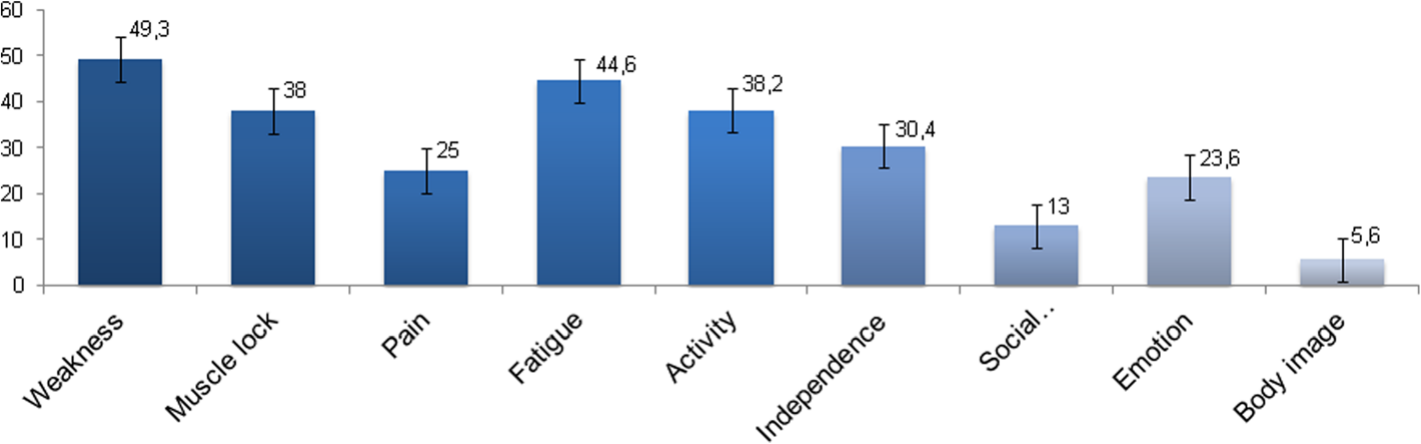
INQoL Domain Scores representing the impact of each impaired domain on patients quality of life; the bars show standard errors. Higher scores indicate the higher impact of disease (*N* = 64)

Figure 2 and Table 6 show the altered domains of quality of life detected by INQoL interview in DM1 patients.

**Table 6:** Characteristics of patients’ and caregiver’s reports at INQoL interview; discrepancy scores are shown in bold (*N* = 64)

INQoL items (range)	Subitems INQoL	Patient’s rating mean (SD)	Caregiver’s rating mean (SD)	Discrepancy mean (caregiver score – patient score)	N° pt. with reduced awareness of illness (%)
1. Weakness (0–19)	*-*	10.1 (5.4)	-	-	
2. Stiffening (0–19)	-	7.4 (6)	-	-	
3. Pain (0–19)	-	5.1 (6)	-	-	
4. Fatigue (0–19)	-	8.9 (5.6)	-	-	
5. Activity (0–18)	-	11.1 (8.3)	10.9 (10.6)	**0.2**	23 (35.9)
6. Independence (0–18)	-	6.2 (5.4)	7.9 (5.6)	1.7*	24 (37.5)
7. Relationships (0–24)	-	6.8 (8.7)	10.1 (11.8)	3.3*	27 (42.2)
8. Emotional	Anxiety (0–6)	2.3 (1.7)	2.8 (1.9)	**0.5**	7 (10.9)
	Depression (0–6)	1.8 (1.7)	1.8 (1.7)	**0.0**	6 (9.3)
	Frustration (0–6)	1.3 (1.7)	1.8 (1.8)	**0.5**	8 (12.5)
	Self-esteem (0–6)	1.1 (1.6)	1.6 (1.7)	**0.5**	7 (10.4)
9. Physical appearance (0–18)	*-*	5.6 (4.6)	-	-	*-*

INQoL total mean (SD) = 19.1 (13.1)

^*^ Statistical significance at *p* < 0.05

According to previous observations [38, 39], also in our analysis the INQoL Total score (19.1) revealed a mild impact of disease disability on quality of life in DM1 patients, probably related to the difficulties in cognition and the lack of interest. By considering the single INQoL Domain Scores, we observed that the Weakness domain had the worst impact (49.3), as well as the Fatigue domain (44.6). Nevertheless, Body Image and Social relationships domains (respectively 5.6 and 13) seemed to be less affected by the disabling effects of disease. Female patients were more likely to refer depressed mood (item 8) than males; no other gender difference were found in INQoL domains analysis (Fig. 2).

### Assessment of disease unawareness

**Fig. 3:**
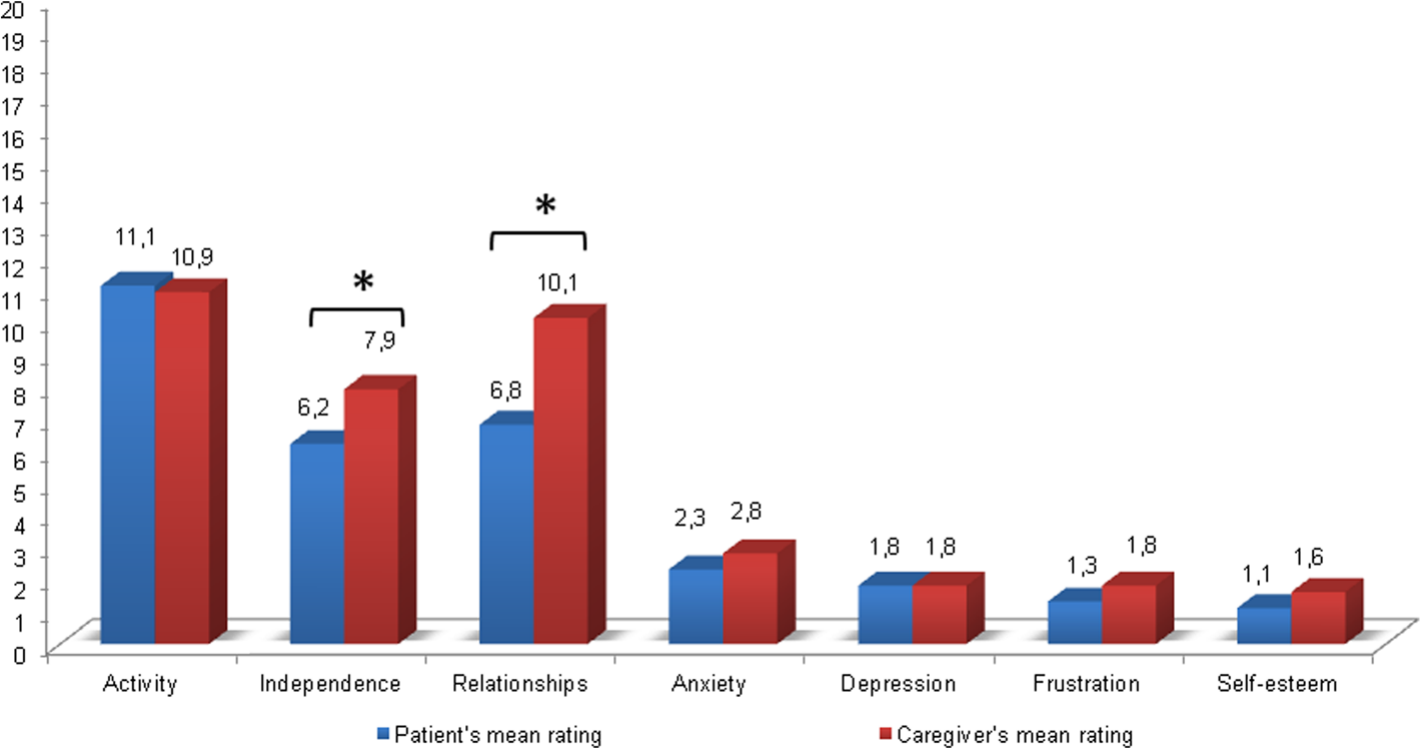
Patients (blue) vs main caregiver (red) mean ratings in INQoL Life Domains (*N* = 64)

The majority of patients were roughly able to describe their behaviours when they were tested by INQoL, although they could not show consistent emotional concern about the consequences of their physical and behavioural symptoms (Fig. 3, Table 6).

**Fig. 4:**
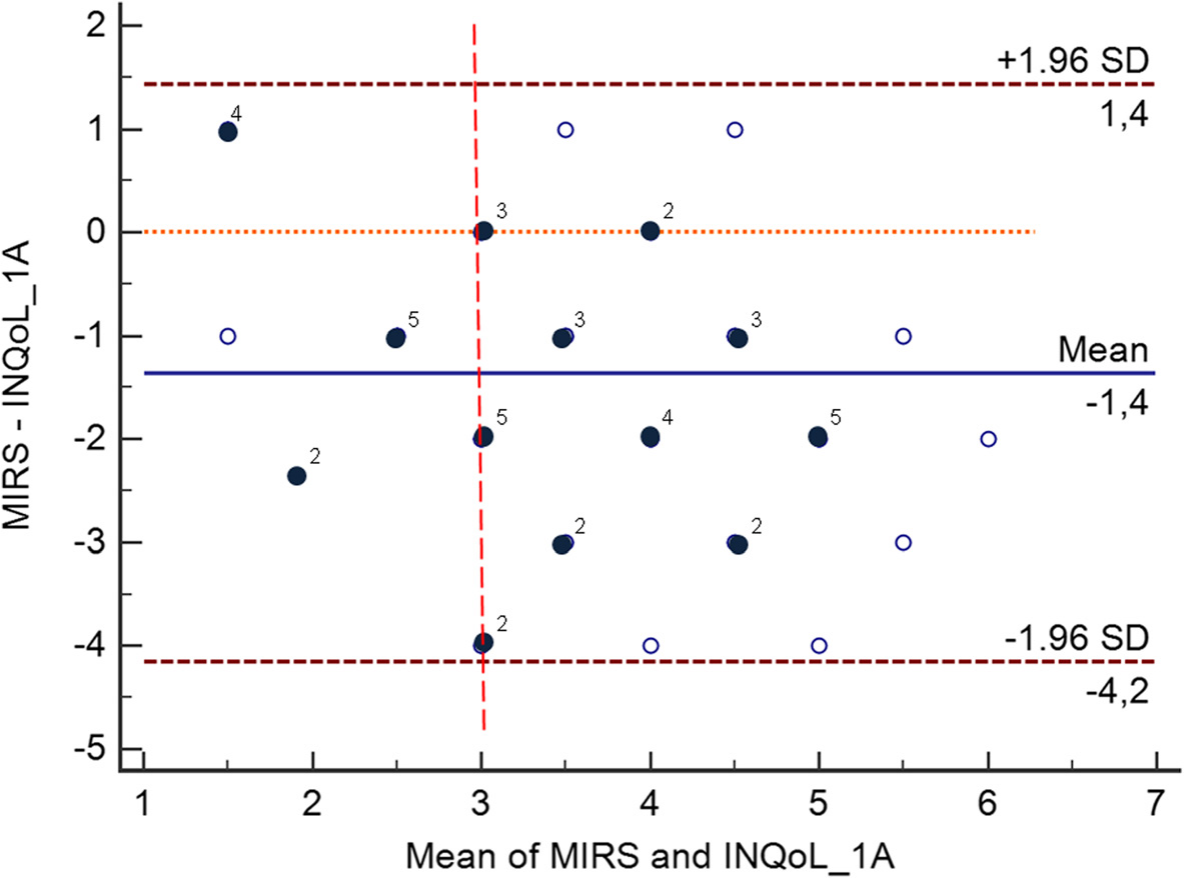
Bland-Altman plot with 95 % limits of agreements for the combined graphical/statistical interpretation of patients’ self evaluation of muscular weakness (INQoL Weakness Domain score) to clinician’s objective muscular evaluation (MIRS) in DM1 patients. The differences between MIRS and INQoL Weakness Domain score are plotted against their mean and the mean difference; light spots indicate single observation, dark spots indicate collinear observations; 95 % confidence limit lines are drawn (brown dotted lines)

Overall, by comparing patients’ self evaluation of muscular weakness (INQoL Weakness Domain score) to clinician’s objective muscular evaluation (MIRS), we found a significant direct correlation between these two measures (*r* = 0.50; *p* < 0.05). However, by using Bland-Altman plot analysis (Fig. 4), we observed a good agreement between patients’ INQoL Weakness score and MIRS only for subjects with a more severe motor impairment (mean of MIRS and INQoL weakness ≥3, Fig. 4). A reduced agreement was instead detected for subjects with a milder muscular phenotype (18.7 %), thus indicating the presence of motor impairment understatement in this subgroup of patients.

Caregivers were predominantly women (63 %, *n* = 41), ranging from 28 to 70 years of age; they were primarily spouses (56.9 %, *n* = 37) or parents (32.3 %, *n* = 21) living at home with the patients.

Internal discrepancies between DM1 patient INQoL self-ratings and main caregiver’s reports were detected in 51.6 % of cases. We observed that patients tended to understate some aspects of their psycho-social difficulties. Notably, statistical analysis revealed a reduced awareness of disease that raised when patients were asked to talk about Life Domains of INQoL, especially Independence (52.4 %) and Social Relationships (47.6 %), (Fig. 3, Table 6) thus suggesting an impairment in self-appraisal of their adaptive behaviours and interaction with the environment.

Notably, normal awareness was detected in INQoL domains concerning the emotional sphere and, overall, a mild occurrence of unawareness was observed about mood alterations (anxiety 19.4 %, depression 16.1 %). No group differences between anosognosic and non anosognosic patients were detected in mood assessment scales (BDI-II *p* = 0.969, STAI-Y2 *p* = 0.787, AES *p* = 0.386).

## Discussion

Although CNS impairment and reduced compliance are well documented, to our knowledge this is the first attempt to investigate disturbances in awareness in DM1 patients, in association with neuropsychological profile and other clinical and demographic variables. Overall, our evidences, rather than a general disease unawareness, support far more unawareness for particular psychosocial and behavioural aspects in DM1 patients.

Since psychological tools for anosognosia evaluation have to be disease-specific, here we proposed a novel neuropsychological protocol to investigate *disease unawareness* in DM1 patients by analysing the discrepancy of the INQoL scores between patient and caregiver’s report [35]. INQoL interview allowed us to detect symptom-specific issues in neuromuscular disorders, usually disregarded by common questionnaires, through the investigation of patient perception. The discrepancy of the INQoL scores was taken into account separately in each domain of every-day-life functioning in order to achieve a characterization of awareness at a single-domain specificity level. Data elaboration revealed that a high percentage (51.6 %) of DM1 patients was unaware of symptoms across different physical and life domains investigated by INQoL interview. In particular, the anosognosia was mostly associated to domains of Independence and Relationships. Despite clear limitations in social functioning, such as work inability or progressive withdrawal from relationships, patients appeared unconcerned about that, as confirmed by caregivers’ reports. Nevertheless, these data could also reflect the effects of patient’s unawareness on the relationship with the main caregiver in coping with illness-related problems.

Interestingly emotional dimensions did not substantially take part to disease unawareness in our sample also for the difficulty, inherent to the nature itself of the administered questions to plenty capture, for both patients and caregivers, emotional reactions to the disease.

From a neuropsychological point of view, most of our patients presented main cognitive impairment in executive and mnesic domains with visuo-spatial involvement, coherently with literature [10, 16, 17]. Patients’ performance suggested that in this sample disease unawareness was related to cognitive dysfunction, specifically with lower scores in visuo-spatial memory, cognitive flexibility, comprehension and conceptualization. Furthermore the neuropsychological tests exploring these cognitive domains, such as CBT and ROCF-copy, showed good sensitivity in discriminating DM1 patients performance vs healthy controls in 76.7 % of cases. We believe that “cognitive indicators” could be useful for future research aimed at defining reliable outcome measures of DM1 neuropsychological involvement and progression.

Some possible methodological limitations of our study, such as sample size discrepancy between patients and controls, or lacking of data on possible impact of family burden on the caregiver, have to be addressed for future studies.

Nonetheless and with the above considerations, anosognosia with a structured profile remains a recurrent feature in DM1, significantly associated to cognitive impairment. Our observations are in line with some theoretical models, such as the *Cognitive Awareness Model* [40] and *Petrified Self Model* [41] that identify self-awareness as a metacognitive function relying on high-order cognitive abilities with consistent structural and functional connectivity with frontal circuitry. Moreover these results are coherent with the hypothesis that DM1 patients present dysfunctions in *social cognition*, that is a set of psychological processes including recognizing common social knowledge and emotional relevance of everyday information coming from the environment [21, 26].

Based on literature evidences, we can hypothesize that anosognosia in DM1 patients is related to brain dysfunction [42]. Several recent neuroimaging studies have implicated various aspects of self-awareness with the frontal lobes and parietal structures [43]. Functional MRI studies have repeatedly observed two large and closely interconnected neural networks, the default mode network (DMN) and the “attention system”, involving respectively medial prefrontal cortex (mPFC), posterior cingulated cortex (pCC), inferior parietal lobule (IPL), lateral and inferior temporal cortex, medial temporal lobe, and dorso-lateral prefrontal cortex (dlPFC), dorsal anterior cingulated cortex, frontal eye fields and extrastriate cortex [44].

Thus it will be suitable to warrant further research to elucidate the mechanisms of anosognosia. Our team is still working on neuroanatomical correlates and functional connectivity of anosognosia by advanced MRI techniques (data not published).

## Conclusions

Finally, gaining a better understanding of anosognosia could improve prognostication, enable timely interventions for DM1 patients, and assist caregivers in early care planning. A cognitive-behavioural and physical exercise intervention trial could be part of the therapeutic strategies aimed to improve quality of life [45]. This is mandatory to prevent detrimental events related to disease progression and to attain specific clinical protocols in preparation of trial readiness.

## Abbreviations

Cat: categorization scoring of WCST
CBT: corsi’s block test
Digit Span: immediate verbal memory test
DM1: myotonic dystrophy type 1
Ep: perseverative errors scoring of WCST
FAB: frontal assessment battery
FAS: phonemic verbal fluency test
INQoL: individualized neuromuscular quality of life questionnaire
MIRS: muscular impairment rating scale
MRI: magnetic resonance imaging
PM47: progressive matrices abstract reasoning test
RAVLT-DR: delayed recall of the rey auditory verbal learning test
RAVLT-IR: immediate recall of the rey auditory verbal learning test
ROCF-copy: copy of the Rey-Osterrieth Complex Figure test
ROCF-DR: delayed recall of the rey-osterrieth complex figure test
ROCF-IR: immediate recall of the rey-osterrieth complex figure test
TMT: trail making test
WCST: Wisconsin card sorting test
